# Audit of Compliance With BURST-BAUS FIX-IT Guidelines for Scrotal Exploration in Suspected Testicular Torsion: A Two-Cycle Quality Improvement Study

**DOI:** 10.7759/cureus.90962

**Published:** 2025-08-25

**Authors:** Haadia Safdar, Ahsan Iftikhar, Nouman Khan, Omar Algurabi, Saif Uddin, Khadeer Abdulkarim, Shanu Sivakumaran, Danial Bajwa, Faisal Ghumman, Javed Burki

**Affiliations:** 1 Urology, Medway National Health Service (NHS) Foundation Trust, Gillingham, GBR; 2 Medical Sciences, Canterbury Christ Church University, Canterbury, GBR; 3 Medicine, City St. George's, University of London, London, GBR; 4 Surgery, Medway National Health Service (NHS) Foundation Trust, Gillingham, GBR

**Keywords:** clinical audit, scrotal orchidopexy, testicular torsion, torsion, urological emergency

## Abstract

Introduction

Testicular torsion is a urological emergency requiring prompt diagnosis and intervention to salvage testicular viability. The British Association of Urological Surgeons (BAUS) and British Urology Researchers in Surgical Training (BURST) released consensus guidelines known as the Finding Consensus for Orchidopexy in Torsion (FIX-IT) study, to standardize scrotal explorations and improve clinical outcomes. This audit conducted at a district general hospital in Kent aimed to evaluate compliance with these guidelines - over two cycles, identifying areas for improvement.

Methods

A retrospective review of operation notes was conducted for all scrotal explorations performed in two distinct audit cycles in 2024. The first cycle, which spanned from January to June 2024, included a total of 16 cases. After completing the first cycle, a review in July 2024 highlighted areas for improvement. Based on this feedback, recommendations for practice improvements were made. The second cycle, conducted from August to December 2024, involved 38 cases, with data collection completed at the end of December. The data gathered for both cycles included the initial incision choice, intraoperative decision-making, fixation technique, closure methods, operation note documentation, and follow-up planning. These domains were then compared to the BURST-BAUS consensus guidelines to evaluate compliance and identify areas for improvement.

Results

A total of 54 scrotal explorations (16 in the first cycle and 38 in the second cycle) were reviewed across both audit cycles. The use of median raphe incisions was universal, with 100% compliance in both cycles. Fixation techniques were fully compliant with the guidelines, with non-absorbable sutures and three-point or four-point fixation being consistently used across both cycles. Closure techniques demonstrated 100% compliance, with all cases employing separate closure of the Dartos and skin layers using continuous and interrupted sutures, respectively, to optimize healing and minimize complications. However, intraoperative decision-making revealed notable divergence from the guidelines, particularly in non-torsion cases where orchidopexy was performed even in the absence of torsion or associated risk factors. In Cycle 1, all non-torsion cases (100%) underwent orchidopexy, whereas in Cycle 2, this was reduced (75%) but still persisted in a significant proportion of cases. Documentation of critical details (such as the degree of torsion and testicular appearance) and follow-up planning remained inconsistent but showed improvement between the two cycles.

Conclusion

The audit highlights substantial improvement between cycles, particularly in reducing inappropriate orchidopexy and planning follow-up. However, documentation gaps and residual guideline non-adherence indicate the need for ongoing education and standardization of scrotal exploration practice.

## Introduction

Testicular torsion is a time-critical surgical emergency caused by the twisting of the spermatic cord, which compromises blood flow to the testis and may result in irreversible ischemic damage if not promptly corrected [1]. Incidence rates of testicular torsion are estimated at 3.8 per 100,000 males under 18 years annually [2]. Salvage rates are closely linked to the duration of ischemia, with the “golden window” for intervention widely accepted as within 4-6 hours of symptom onset [3,4]. The testicular salvage rate is around 90% if the spermatic cord is detorsed within six hours of symptom onset. However, this rate drops to 50% after 12 hours and falls below 10% after 24 hours [5].

The diagnosis of acute testicular torsion is mainly determined by clinical signs and symptoms, although Doppler ultrasound may be helpful in certain cases [6]. However, it is important to note that testicular torsion can only be definitively excluded through surgical exploration [7].

Despite its urgency, the intraoperative management of suspected testicular torsion lacks global standardization. Decision-making regarding fixation, exploration of the contralateral testis, and management when no torsion or alternative pathology is found has varied historically [8]. The recent Finding Consensus for Orchidopexy in Torsion (FIX-IT) statement from the British Urology Researchers in Surgical Training (BURST) and the British Association of Urological Surgeons (BAUS) provides expert consensus on best practices for patients undergoing emergency scrotal exploration [9].

The key guideline recommendations from the FIX-IT study include the following [9].

Consent

Thoroughly discuss potential risks (e.g., orchidectomy and infection) and do not offer a prosthesis during initial surgery.

Anesthetic assessment

Proceed with exploration even if suspicion of torsion decreases under anesthesia.

Initial incisions

Both craniocaudal median raphe and transverse incisions are acceptable, with no clear preference established.

Intraoperative decision-making

If torsion is present and the testis is still viable, untwist and fix, including contralateral fixation. For an ischemic testis, attempt warm gauze application and reassess; orchidectomy should be performed if the testis is unviable. If torsion is absent, fixation is not needed unless a bell clapper deformity is present.

Testicular fixation

Use sutureless (Dartos pouch) or sutured fixation in children; adults should have sutured fixation with non-absorbable sutures.

Closure

The Dartos layer should be closed separately from the skin. For the Dartos layer, a continuous suture is preferred. For the skin, interrupted sutures are preferred.

Intraoperative photography

Avoid routine medical photography for documenting testis appearance.

Operation notes

Clearly document fixation techniques, suture details, and intraoperative findings.

Post-operative follow-up

Tailor follow-ups based on findings, emphasizing recovery monitoring, fertility preservation, and prosthesis discussions when needed.

Logistical considerations

Minimize delays to surgery and ensure any trained surgeon can perform the procedure to expedite care.

This paper presents a two-cycle audit assessing compliance with these guidelines, aiming to identify gaps, improve practice, and contribute to wider discussions on standardizing scrotal exploration for suspected torsion.

## Materials and methods

A retrospective analysis of operative notes from scrotal explorations performed at a district general hospital was conducted in two audit cycles: Cycle 1: January 2024-June 2024 (n = 16) and Cycle 2: August 2024-December 2024 (n = 38). Inclusion criteria were all male patients undergoing scrotal exploration for suspected testicular torsion. All these scrotal explorations were conducted by urology trainees or registrars.

In July 2024, between the two cycles, the team actively attempted to improve compliance with the BURST-BAUS guidelines by conducting educational sessions focusing on the importance of proper decision-making in non-torsion cases and feedback meetings, ensuring adherence to the guidelines on fixation in non-torsion cases. While these measures were implemented during July, full compliance was not immediately achieved, and improvements were observed only after the feedback process was incorporated into practice, leading to better adherence in Cycle 2. Data were extracted from operation notes using a structured proforma focusing on compliance with BURST-BAUS domains (Table 1).

**Table 1:** Domains assessed in this audit As per recommendations given in the Finding Consensus for Orchidopexy in Torsion (FIX-IT) study [9], which is an open-access article distributed under the terms and conditions of CC BY-NC 4.0 license.

Assessed domains	
1	Initial incision type
2	Intraoperative decision-making: identifying whether torsion, no torsion/no pathology, or alternative pathology was found, and subsequent management (orchidopexy, orchidectomy)
3	Fixation technique: including suture type and fixation approach
4	Closure technique: separate Dartos and skin closure
5	Documentation quality: presence/absence of key details (degree of torsion, testis appearance)
6	Post-operative follow-up: documented plan according to guideline recommendations

The primary outcome was the proportion of cases compliant with guidelines in each domain. Secondary outcomes included a comparative analysis between the two cycles to assess improvements post-initial audit feedback.

## Results

Table 2 summarizes the demographic details of the cases included in the audit. In Cycle 1 (January to June 2024), 16 cases were reviewed, with a median patient age of 16.43 years. In Cycle 2 (August to December 2024), the number of cases increased to 38, and the median age of patients rose to 17.44 years.

**Table 2:** Demographics and overview of cases

Cycle	Number of cases (n)	Median age
Cycle 1 (Jan–Jun 2024)	16	16.43 years
Cycle 2 (Aug–Dec 2024)	38	17.44 years

Initial incision type

All scrotal explorations performed utilized a median raphe/midline scrotal incision, ensuring a 100% compliance rate. The consensus suggests both craniocaudal median raphe and transverse incisions (over the affected testis) are acceptable. Table 3 outlines the incision types.

**Table 3:** Compliance with incision type

Cycle	Incision type	Compliance
Cycle 1	Median raphe	100% (16/16)
Cycle 2	Median raphe	100% (38/38)

Intraoperative decision-making

In terms of intraoperative decision-making, the audit revealed notable differences between the two cycles. Table 4 below provides the summarized results.

**Table 4:** Intraoperative decision-making

Cycle	Torsion	No torsion, no other pathology	Other pathology (appendix testis/epididymitis)
Cycle 1	5	4	7
	3 viable – orchidopexy; 2 non-viable - orchidectomy and contralateral orchidopexy	4/4 orchidopexy (100%)	7/7 orchidopexy (100%)
Cycle 2	7	20	11
	6 viable – orchidopexy; 1 non-viable - orchidectomy and contralateral orchidopexy	15/20 orchidopexy (75%)	8/11 orchidopexy (72%)

**Figure 1:**
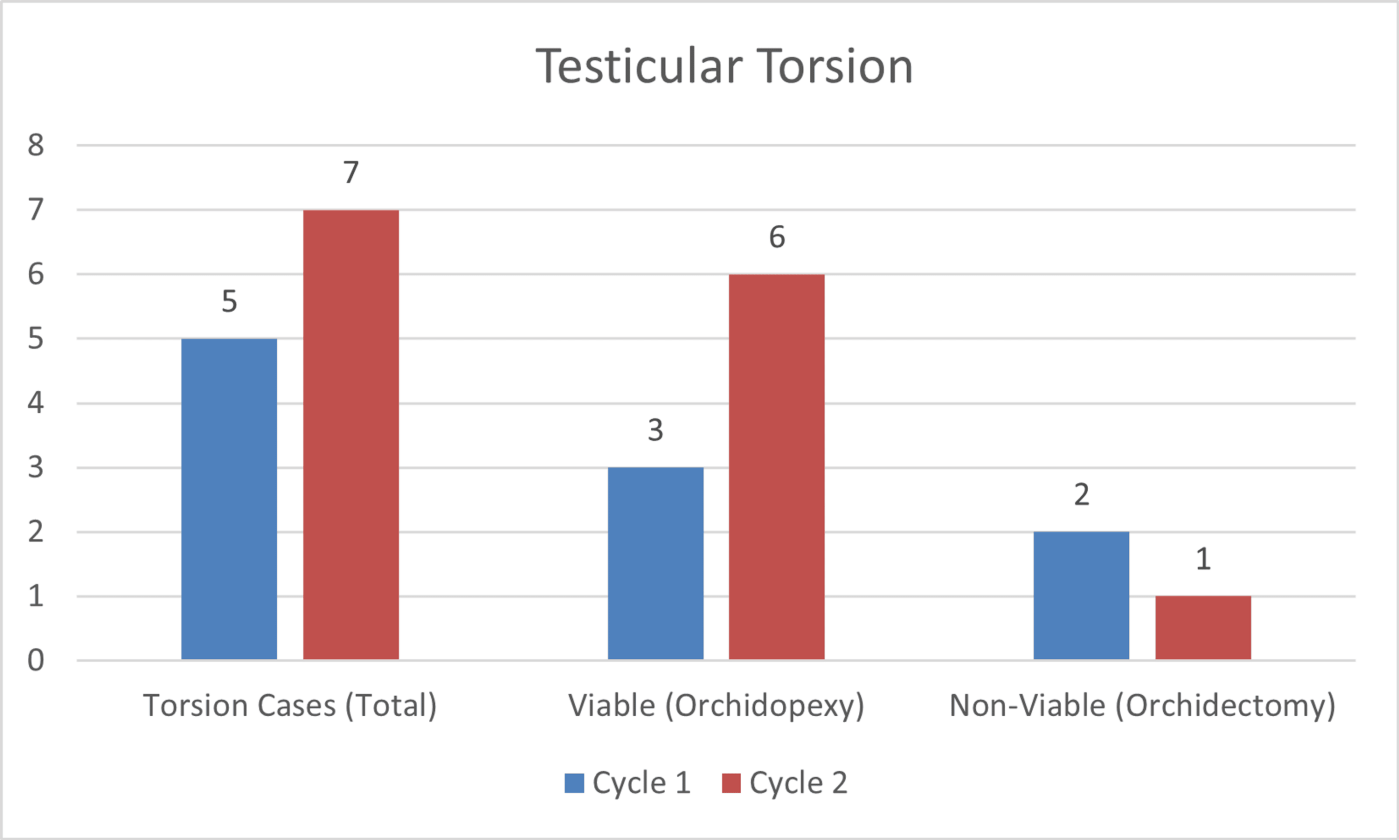
Management of testicular torsion cases in both cycles

For torsion cases, in Cycle 1 (January-June 2024), five out of 16 cases (31%) involved torsion, with three viable testes undergoing orchidopexy and two non-viable testes requiring orchidectomy and contralateral orchidopexy. In Cycle 2 (August-December 2024), seven out of 38 cases (18%) were torsion cases, with six viable testes receiving orchidopexy and one non-viable testis requiring orchidectomy and contralateral orchidopexy. The following chart outlines the results (Figure 1).

Regarding non-torsion cases, in Cycle 1, four out of 16 cases were found to have no torsion or no other pathology, and 4/4 (100%) underwent inappropriate fixation, while seven out of 16 cases with other pathologies, such as inflamed or torted appendix testis/hydatid of Morgagni or epididymitis, were also all fixed in addition to relevant procedures (excision of the appendix testis). These fixations go against the recommended guidelines, as they should not have been fixed.

In Cycle 2, 20 out of 38 cases had no torsion or no other pathology found, 15/20 (75%) were inappropriately fixed, and 11 out of 38 cases with other pathologies had fixation performed in eight of them - 8/11 (72%). These findings highlight the persistence of inappropriate fixation in non-torsion cases, though there was a slight reduction in unnecessary interventions from Cycle 1 (100% in both cases) to Cycle 2 (75% and 72%, respectively).

**Figure 2:**
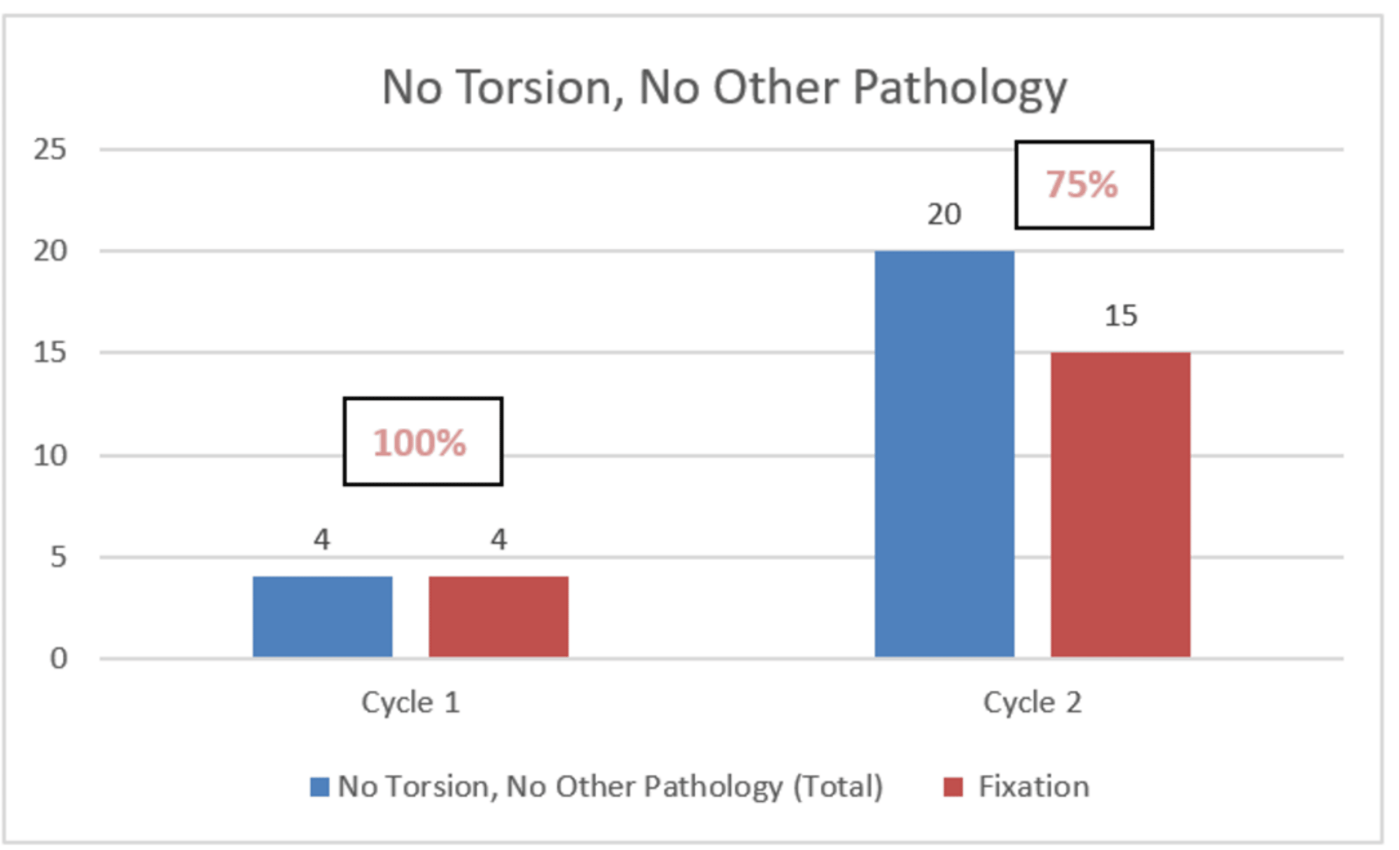
Bar chart showing fixation (orchidopexy) in cases where no other pathology was found Non-compliance shown in percentage (improved from 100% in the first cycle to 75% in the second cycle)

**Figure 3:**
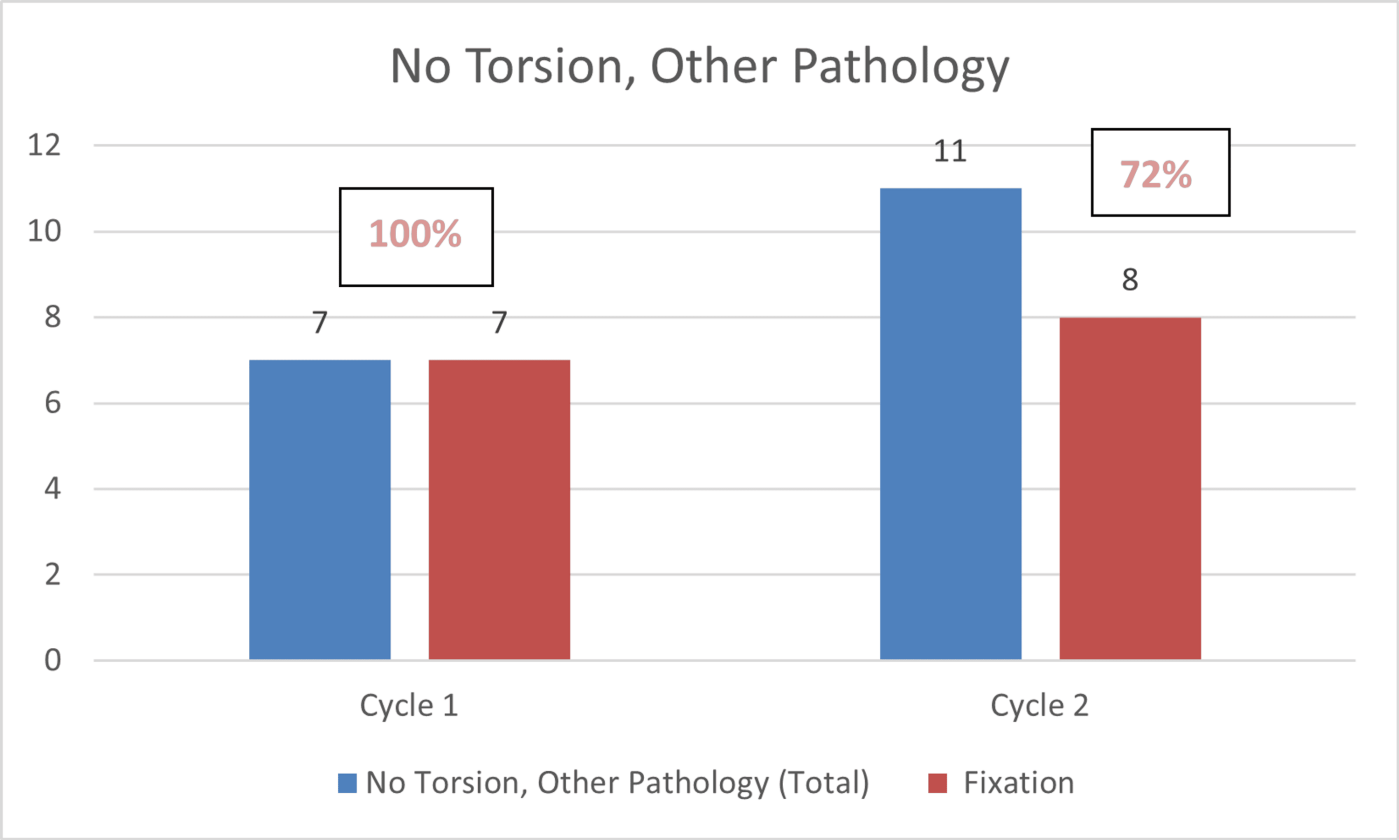
Bar chart showing fixation (orchidopexy) in cases where pathology other than torsion was found Non-compliance shown in percentage (improved from 100% in the first cycle to 72% in the second cycle)

The charts below highlight these findings (Figures 2, 3).

Fixation technique

In both Cycle 1 and Cycle 2, there was 100% compliance with the use of non-absorbable sutures for testicular fixation, specifically polypropylene 3/0 and 4/0. Polypropylene is favored for its strength, durability, and minimal tissue reactivity, ensuring secure fixation of the testis and preventing re-torsion. All cases also adhered to the recommended three-point fixation technique, which is in line with the BURST-BAUS guidelines (shown in Table 5).

**Table 5:** Fixation technique compliance

Cycle	Non-absorbable suture	3-point/4-point fixation	Compliance
Cycle 1	Yes	Yes	100% (16/16)
Cycle 2	Yes	Yes	100% (38/38)

Closure technique

The closure technique for scrotal explorations adhered to the BURST-BAUS guidelines (as shown in Table 6), with the Dartos and skin layers being closed separately to optimize healing and minimize complications. For the Dartos layer, a continuous suture technique was utilized, providing a secure, uniform closure while promoting faster healing and reducing the risk of hematoma formation. For skin closure, interrupted sutures were primarily used. Some surgeons used simple interrupted sutures while others opted for vertical mattress sutures, which provide additional tissue eversion and better hemostasis.

**Table 6:** Closure technique compliance

Cycle	Closure technique	Compliance
Cycle 1	Dartos and skin closed separately using continuous and interrupted sutures	100% (16/16)
Cycle 2	Dartos and skin closed separately using continuous and interrupted sutures	100% (38/38)

Operation note documentation

The documentation of fixation techniques was consistent throughout the process. However, the degree of torsion and assessments of viability were poorly documented in the first cycle, with better improved documentation noted in the second cycle. Overall, improvement was noted across the two cycles (as shown in Table 7).

**Table 7:** Documentation of key intraoperative findings

Cycle	Documentation of intraoperative findings	Compliance
Cycle 1	Inconsistent	80% (13/16)
Cycle 2	Improved	97% (37/38)

Follow-up planning

Follow-up planning showed notable improvement between the two cycles (Table 8). In Cycle 1, follow-up was poorly managed, with four out of five torsion cases lacking a formal follow-up plan. In contrast, Cycle 2 demonstrated significant progress, with proper follow-up plans established for all torsion cases. Additionally, patients in the non-torsion group were appropriately discharged without the need for a routine follow-up.

**Table 8:** Follow-up planning compliance

Cycle	Follow-up plan	Compliance
Cycle 1	Poorly documented	50% (8/16)
Cycle 2	Well documented (clear discharge for non-torsion cases and appropriate follow-up for torsion cases)	90% (34/38)

## Discussion

This audit aimed to evaluate and improve compliance with the BURST-BAUS consensus guidelines on scrotal exploration for suspected testicular torsion. Testicular torsion remains a surgical emergency with significant implications for fertility and endocrine function if not timely and appropriately managed [10]. While prompt exploration remains the standard of care, uniformity in intraoperative decision-making, especially regarding fixation practices and documentation, is essential for optimizing outcomes [11].

Initial incision was consistently performed via the median raphe approach, allowing access to both testes without creating separate incisions. This is in full alignment with BURST-BAUS recommendations and global best practice standards [12].

A national survey conducted by the Paediatric Surgical Trainee Research Network (PSTRN) and the BURST assessed the surgical management of torted and non-torted testes and revealed that most surgeons refrained from performing suture fixation when torsion was not identified. For alternative diagnoses like testicular appendage torsion, epididymitis, or a normal testis, only 26%-31% preferred anatomical restoration with tunica vaginalis (TV) closure, while a majority (53%-66%) favored non-suture methods such as leaving the TV open, creating a Dartos pouch, or employing Jaboulay eversion. However, when a bell clapper deformity was identified, 69% proceeded with sutured fixation [13].

Previous literature recommends polypropylene non-absorbable as the first line; however, the usage of prolonged absorbable sutures (e.g., polydioxanone) may also be considered to reduce the risk of potential chronic scrotal pain or micro-abscesses relative to their non-absorbable counterparts. Presently, there is no clear consensus on suture size, as this may depend on factors such as testicular volume and the appropriate clinical judgement.

A systematic review by Moore et al. evaluating 182 patients showed significant variability in operative technique. No consensus was achieved between fixation technique, suture characteristics, contralateral orchidopexy, and the evidence behind fixation of non-torted testes [14].

Additionally, a UK study investigating techniques again highlighted bilateral orchidopexy for confirmed torsion of viable testes in 29 respondents; however, less commonly, 66% used three-point fixation, 57% employed polyglactin 910 sutures, and 38% performed synchronous procedures (e.g., Jaboulay procedure or appendix testis excision). Notably, 31% still employed a unilateral orchidopexy approach despite newer guidelines suggesting otherwise [15].

A survey by the Annals of the Royal College of Surgeons (RCS) England evaluated 340 responses from 83 institutions and found predominantly sutured fixations were preferred in 74% of cases, with a sutureless Dartos approach commonly undertaken by pediatric fixations (37%). Sutureless approaches were also used in 53%-66% of non-torsion diagnoses, with sutured fixations used in 69% of cases, with anatomical risk factors like “bell clapper” deformities exhibiting 69% [13].

Dartos layers are recommended to be closed separately from the skin, with a continuous suture technique being preferred. Scrotal compression is recommended in the post-operative period (e.g., via scrotal support). A 2012 retrospective case series recommended the Dartos to be closed with 3/0 polyglactin sutures continuously, with scrotal skin edges approximated with interrupted absorbable sutures. In the 12 cases where such techniques were employed, follow-up within two years showed no instance of re-torsion, suggesting this as a viable method of closure to prevent the potential of repeat operations [16].

A 2017 study looking to improve operative note documentation evaluated 101 operative notes against 19 standards within the 2014 RCS guidelines. Strategies such as clinician education, aide memoires at computer terminals, and revision of surgical proformas provided a dramatic improvement within the second loop. Crucial components often overlooked or missed out consisted of the time of procedure, complications as a result, anticipated and true blood loss, and details of venous thromboembolism (VTE) prophylaxis.

Similarly, another audit in 2014 showed that 20% of all operative notes contained parts that were illegible or incomplete or included difficult-to-decipher abbreviations [17]. Similar audits globally and across specialties displayed similar findings, with most not meeting operative note guidelines set [18,19].

Appropriate operative documentation should be employed, as this may negatively impact post-operative care, pre-discharge, and follow-up planning. Detailing fixation technique, suture characteristics including size and type, presence (±degree) of torsion, appearance, other pathologies noted, and the extent of ischemia are torsion-specific variables that should be thoroughly documented to communicate with other specialists and inform subsequent patient care. Individual patient characteristics and intraoperative findings may influence later investigation, but a standardized approach to documentation may make the overall process more streamlined as a result.

## Conclusions

This two-cycle audit highlights a marked improvement in adherence to BURST-BAUS guidelines following initial feedback, particularly in areas such as follow-up planning and the reduction of unnecessary orchidopexies. The inclusion of a substantial case series and structured comparison aligned with expert consensus strengthens the findings, while the diverse range of participating surgeons reflects real-world practice variability. Nonetheless, the retrospective design and reliance on existing documentation pose inherent limitations, potentially underestimating compliance due to incomplete records. Additionally, the audit did not explore contextual factors such as surgeon experience or perceived intraoperative risks, which may influence deviations from guidelines. Persistent gaps, especially in the documentation of torsion details and the continued use of fixation in non-torsion cases, indicate the need for further interventions. These should include regular re-auditing, targeted training, and implementation of standardized operative note templates to promote full compliance and ultimately enhance the quality and safety of care for patients undergoing scrotal exploration.
